# Development of nintedanib nanosuspension for inhaled treatment of experimental silicosis

**DOI:** 10.1002/btm2.10401

**Published:** 2022-09-19

**Authors:** Luisa Helena Andrade da Silva, Juliana Borges Vieira, Marianna Ribeiro Cabral, Mariana Alves Antunes, Daiheon Lee, Fernanda Ferreira Cruz, Justin Hanes, Patricia Rieken Macedo Rocco, Marcelo Marcos Morales, Jung Soo Suk

**Affiliations:** ^1^ Laboratory of Pulmonary Investigation Carlos Chagas Filho Institute of Biophysics, Federal University of Rio de Janeiro Rio de Janeiro Brazil; ^2^ Rio de Janeiro Innovation Network in Nanosystems for Health – NanoSAÚDE/FAPERJ Rio de Janeiro Brazil; ^3^ Center for Nanomedicine at the Wilmer Eye Institute, Johns Hopkins University School of Medicine Baltimore Maryland USA; ^4^ Department of Ophthalmology Johns Hopkins University School of Medicine Baltimore Maryland USA; ^5^ Department of Chemical and Biomolecular Engineering Johns Hopkins University Baltimore Maryland USA; ^6^ Laboratory of Cellular and Molecular Physiology Carlos Chagas Filho Biophysics Institute, Federal University of Rio de Janeiro Rio de Janeiro Brazil

**Keywords:** localized treatment, lung function, nanosuspension, pulmonary fibrosis, tyrosine kinase inhibitor

## Abstract

Silicosis is an irreversible and progressive fibrotic lung disease caused by massive inhalation of crystalline silica dust at workplaces, affecting millions of industrial workers worldwide. A tyrosine kinase inhibitor, nintedanib (NTB), has emerged as a potential silicosis treatment due to its inhibitory effects on key signaling pathways that promote silica‐induced pulmonary fibrosis. However, chronic and frequent use of the oral NTB formulation clinically approved for treating other fibrotic lung diseases often results in significant side effects. To this end, we engineered a nanocrystal‐based suspension formulation of NTB (NTB‐NS) possessing specific physicochemical properties to enhance drug retention in the lung for localized treatment of silicosis via inhalation. Our NTB‐NS formulation was prepared using a wet‐milling procedure in presence of Pluronic F127 to endow the formulation with nonadhesive surface coatings to minimize interactions with therapy‐inactivating delivery barriers in the lung. We found that NTB‐NS, following intratracheal administration, provided robust anti‐fibrotic effects and mechanical lung function recovery in a mouse model of silicosis, whereas a 100‐fold greater oral NTB dose given with a triple dosing frequency failed to do so. Importantly, several key pathological phenotypes were fully normalized by NTB‐NS without displaying notable local or systemic adverse effects. Overall, NTB‐NS may open a new avenue for localized treatment of silicosis and potentially other fibrotic lung diseases.

## 1 INTRODUCTION

Silicosis is an occupational lung disease caused by continuous inhalation of crystalline silica microparticles, which affects construction, mining, and industry workers.^1^, ^2^, ^3^ It is estimated that more than 2 million U.S. workers are under continuous exposure to silica at workplaces.^3^, ^4^ Silica microparticles, upon deposition in the alveolar sacs, induce pro‐inflammatory response, progressive fibrosis, and irreversible granuloma formation in the lung parenchyma, thereby gradually compromising the pulmonary function.^1^, ^5^ As a result, this devastating disease presents high incapacitation rates^3^ while there is no cure other than lung transplantation, a procedure with limited availability due to lack of suitable donor organs.^1^, ^6^

The oral formulation of a tyrosine kinase inhibitor, nintedanib (NTB; OFEV®), is clinically used for treating other fibrotic lung diseases, including idiopathic pulmonary fibrosis (IPF) and chronic interstitial lung disease, to mitigate lung function decline and the risk of pulmonary exacerbation.^7^ NTB is also under a phase II clinical trial to evaluate its therapeutic benefits in patients with occupational pneumoconiosis (NCT0461014). NTB acts by blocking the fibroblast growth factor receptor‐1 and the platelet‐derived growth factor receptor, thus disrupting downstream signaling cascades that promote proliferation of fibroblasts/myofibroblasts and collagen deposition.^7^, ^8^, ^9^ In addition, NTB inhibits *Src* pathway in silica‐activated macrophages in vitro^9^ and in lung fibrosis in vivo^10^ and, in turn, thwarts the expression of fibrogenic mediators, such as transforming growth factor (TGF)‐β.^10^ To this end, NTB may pose a potential therapeutic option for treating patients with silicosis and/or provide a bridge to future lung transplant.

However, it is important to note that a significant fraction, or even a majority, of orally administered drugs are lost by the first pass effect and the drug amount absorbed into the systemic circulation is shared by different organs.^11^, ^12^ It has been demonstrated that only one‐thousandth of orally administered NTB is found in mouse lungs.^13^ Furthermore, a more recent pharmacokinetic study revealed that only a small fraction of the orally administered yet lung‐partitioned NTB reached epithelial airway surface, the therapeutically relevant compartment within the silicotic lungs.^12^ Thus, a very large and frequent oral NTB dosage is likely required to achieve an effective therapeutic window in the lung, leading to systemic safety concerns and economic burden. Indeed, oral NTB treatment is often associated with gastrointestinal adverse events, which results in discontinuation of its uses among IPF patients.^7^, ^14^, ^15^

We thus sought to develop a NTB formulation that could be administered locally via inhalation to provide a clinically relevant drug concentration in the lung, while minimizing the dose and systemic drug exposure, as well as potential adverse events.^11^, ^12^ Specifically, we engineered a nanocrystal‐based nanosuspension (NS) formulation of NTB (NTB‐NS), surface‐stabilized with adsorptive nonadhesive polymer coatings, and evaluated its therapeutic efficacy in a mouse model of silicosis, following direct administration into the lungs via intratracheal instillation.

## 2 RESULTS AND DISCUSSION

### 2.1 Formulation and characterization of NTB‐NS

**FIGURE 1:**
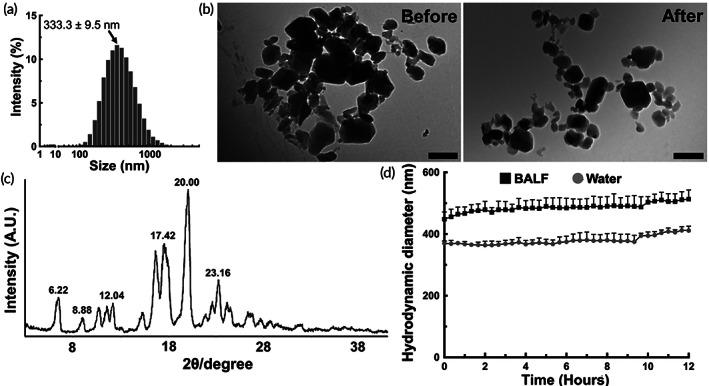
Physicochemical properties of NTB‐NS are preserved after nebulization or in a physiologically relevant lung environment. (a) Hydrodynamic diameters of NTB‐NS. (b) Representative transmission electron micrographs of NTB‐NS before (left) and after (right) aerosolization via a nebulizer. Scale bars = 500 nm. (c) X‐ray diffraction crystallography of NTB‐NS. (d) Colloidal stability of NTB‐NS in water (gray circles) and in mouse BALF (black squares). Data represents mean ± SD (n = 3 independent samples). NTB‐NS, nanosuspension formulation of nintedanib

NTB in a free‐base form presents low aqueous solubility, which reduces its bioavailability in the physiological lung environment.^11^ Thus, we sought to develop a formulation that could be stably dispersed in aqueous solutions to be directly administered into the lung. Specifically, we engineered a nanocrystal‐based NS formulation of NTB (i.e., NTB‐NS) by varying the variables to yield particles with nonadhesive surface coatings and nanoscale dimensions to potentially minimize mucus entrapment and macrophage uptake, following inhaled administration.^16^, ^17^, ^18^, ^19^, ^20^, ^21^ The ability to do so increases the therapeutically available drug concentration in the lung.^22^, ^23^, ^24^ We tested concentration ranges of poloxamer 407 (i.e., Pluronic F127) and NTB (Figure S1A‐B), where F127 endows the formulation with nonadhesive surface coating via physical adsorption,^25^, ^26^ and determined a formulation prepared at 1% F127 and 45 mg/mL NTB to be our lead formulation. The NTB‐NS exhibited polygonal structure (Figure S1C) and hydrodynamic diameters of 333.3 ± 9.5 nm with polydispersity indices of 0.21 ± 0.02 (Table 1, Figure 1a). We also found that approximately 90% of the initial NTB amount was loaded into the final NS formulation (Table 1, Figure 1a), which was markedly greater than encapsulation efficiencies of NTB enabled by other commonly used delivery platforms, such as liposomes (34%) and polymeric nanoparticles (5%).^27^

**TABLE 1:** Physicochemical characterization of NTB‐NS

Hydrodynamic diameter (*Z*‐Ave) [nm]	Polydispersity index	ζ‐potential [mV]	Encapsulation efficiency [%]
333.3 ± 9.5	0.21 ± 0.02	8.1 ± 0.5	89.5 ± 2.2

*Note*: Data represents mean ± SD (n = 3 independent samples).

*Abbreviation*: NTB‐NS, nanosuspension formulation of nintedanib.

We then confirmed via transmission electron microscopy that the size and morphology of NTB‐NS were retained after being aerosolized via a vibrating mesh nebulizer (Figure 1b), a clinically used inhalation device. X‐ray diffraction analysis revealed that NTB‐NS existed as crystalline solids with refraction angles (2*θ* scale) of 6.22°, 8.88°, 12.04°, 17.42°, 20°, and 23.16° (Figure 1c). Unlike in water, hydrodynamic diameters of NTB‐NS slightly increased immediately upon incubation in bronchoalveolar lavage fluid (BALF) at 37°C but the particle size remained unchanged at least up to 12 hours (Figure 1d), underscoring excellent colloidal stability in a physiologically relevant lung environment. Of note, NTB‐NS was prepared using aseptic technique, autoclaved utensils, and sterile‐filtered solutions inside a laminar flow hood to avoid bacterial contamination, etc., and the sterility was confirmed by the absence of microbial colonies following a 1‐week inoculation on tryptic soy agar plates (Figure S1D). For long‐term storage as a powder form, NTB‐NS was lyophilized in presence or absence of a disaccharide‐based lyoprotectant, and subsequently, the lyophilized NTB‐NS was rehydrated for physicochemical characterization. We found that lyophilization in 3% sucrose did not perturb the particle size whereas significant aggregation was observed when NTB‐NS was lyophilized in 3% trehalose or without any lyoprotectant (Figure S2).

### 2.2 In vivo safety of locally administered NTB‐NS in the lungs of healthy animals

To evaluate preclinical safety, we dosed healthy C57BL/6 mice with NTB‐NS via intratracheal instillation^28^ to ensure reliable dose‐response assessment, since a fraction of nebulized drugs is deposited in the oropharynx during the transit to the deeper lung.^29^ We selected NTB‐NS doses to be tested by benchmarking prior studies demonstrating that oral administration of 100 mg/kg NTB rendered approximately 2.5 μg of the drug available in mouse lungs,^13^, ^30^ which roughly correspond to a local dose of 0.1 mg/kg NTB. We thus treated animals in different groups at three incrementing doses of 0.01 (i.e., 10‐fold lower), 0.1, and 1 (i.e., 10‐fold higher) mg/kg. Control mice were identically treated with the vehicle used for NTB‐NS preparation and administration (i.e., ultrapure water).

**FIGURE 2:**
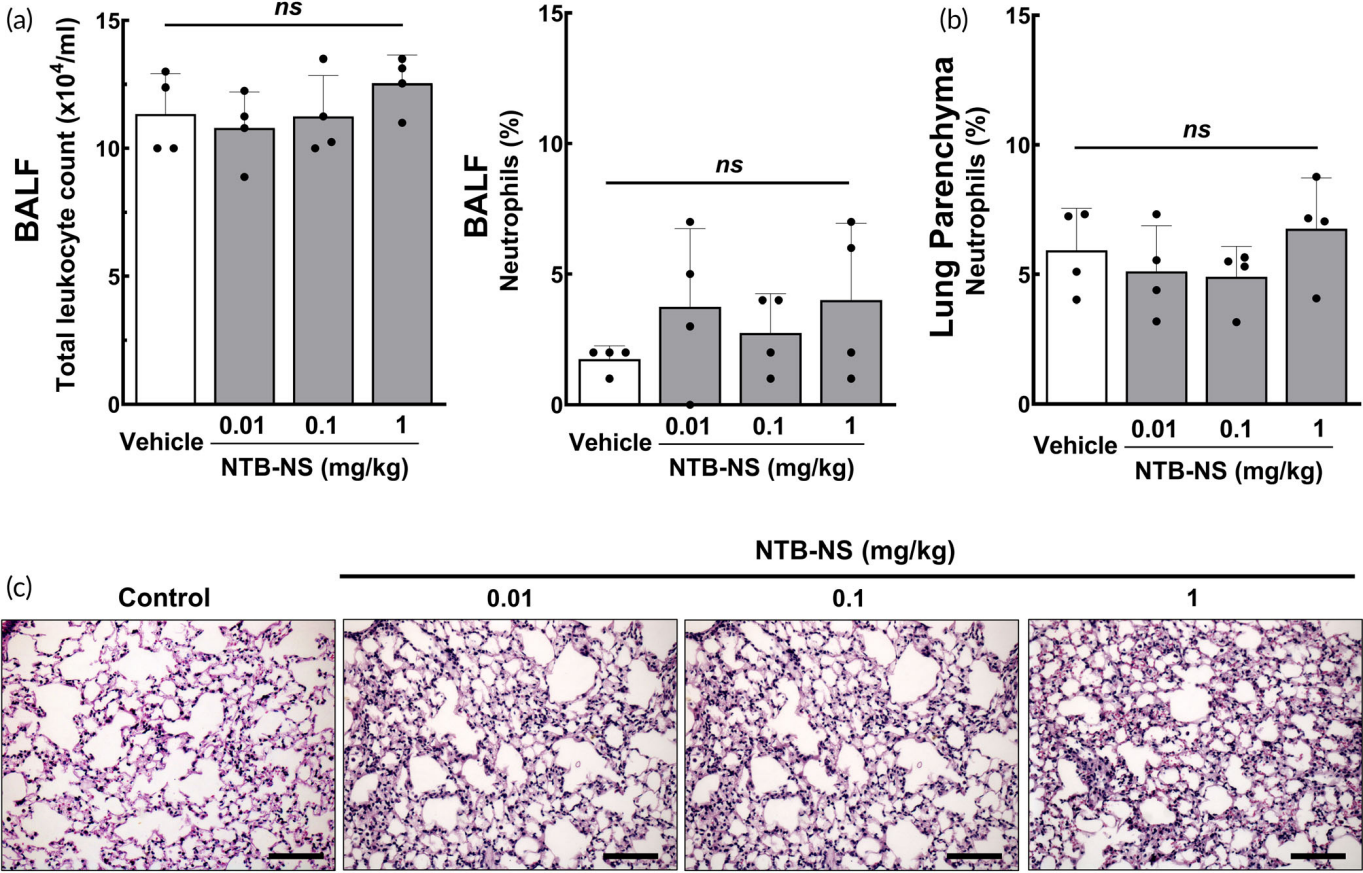
Intratracheally administered NTB‐NS does not induce undesired pro‐inflammatory response in the lungs of healthy mice. (a) Total leukocyte counts and percentage of neutrophils in BALF. (b) Percentage of neutrophils in lung parenchyma. Bars represent mean *±* SD (n = 4 mice per group). The differences are not statistically significant as indicated (ns; one‐way ANOVA followed by a Tukey post hoc test). (c) Representative histological images of lung parenchyma. Scale bars = 200 μm. NTB‐NS, nanosuspension formulation of nintedanib

We first confirmed that body temperature (Figure S3A) and weight (Figure S3B) were unchanged 24 hours after the administration regardless of the NTB‐NS dose, suggesting that there was no significant acute systemic toxicity. We then harvested BALF from individual animals to analyze cellularity for local safety assessment. The differences in the total number of leukocytes and percentage of neutrophils (Figure 2a) were not significant between animals that received vehicle (i.e., ultrapure water) and those that received different doses of NTB‐NS. We also harvested lung tissues for histological analysis and found that the percentage of neutrophils in the lung parenchyma was not elevated by intratracheal NTB‐NS instillation (Figure 2b,c). This observation suggests that local administration of NTB‐NS does not elicit acute adverse events in the healthy mouse lungs, presumably attributed to its preparation in an aseptic condition and to the use of the materials generally regarded as safe only (i.e., F127) for preparing the formulation.^31^

### 2.3 In vivo therapeutic efficacy of locally administered NTB‐NS in the lungs of silicotic animals

We next investigated whether intratracheally administered NTB‐NS could attenuate the progression of silica‐induced fibrosis in vivo. Treatments were commenced 15 days after the induction of silicosis by a single intratracheal instillation of silica at a dose of 800 mg/kg. We have previously demonstrated that pulmonary fibrosis is established at this time point and stably retained at least up to 30 days post‐instillation.^28^ Specifically, we treated silicotic animals with NTB‐NS via intratracheal administration at a dose of 0.1 or 1 mg/kg every 72 hours up to six overall doses, while animals in a separate group received daily oral doses of NTB Esylate (NTB‐Esy) at 100 mg/kg for 18 days (Figure S4). Of note, daily oral treatments with NTB at 100 mg/kg was previously shown to significantly reduce the fibrotic score in the lungs of silicotic animals, but the model was established with a markedly lower silica dose (2.5 mg/mouse)^8^ compared to our study where individual animals were intratracheally instilled with an 8‐fold greater silica dose (i.e., 20 mg/mouse).

**FIGURE 3:**
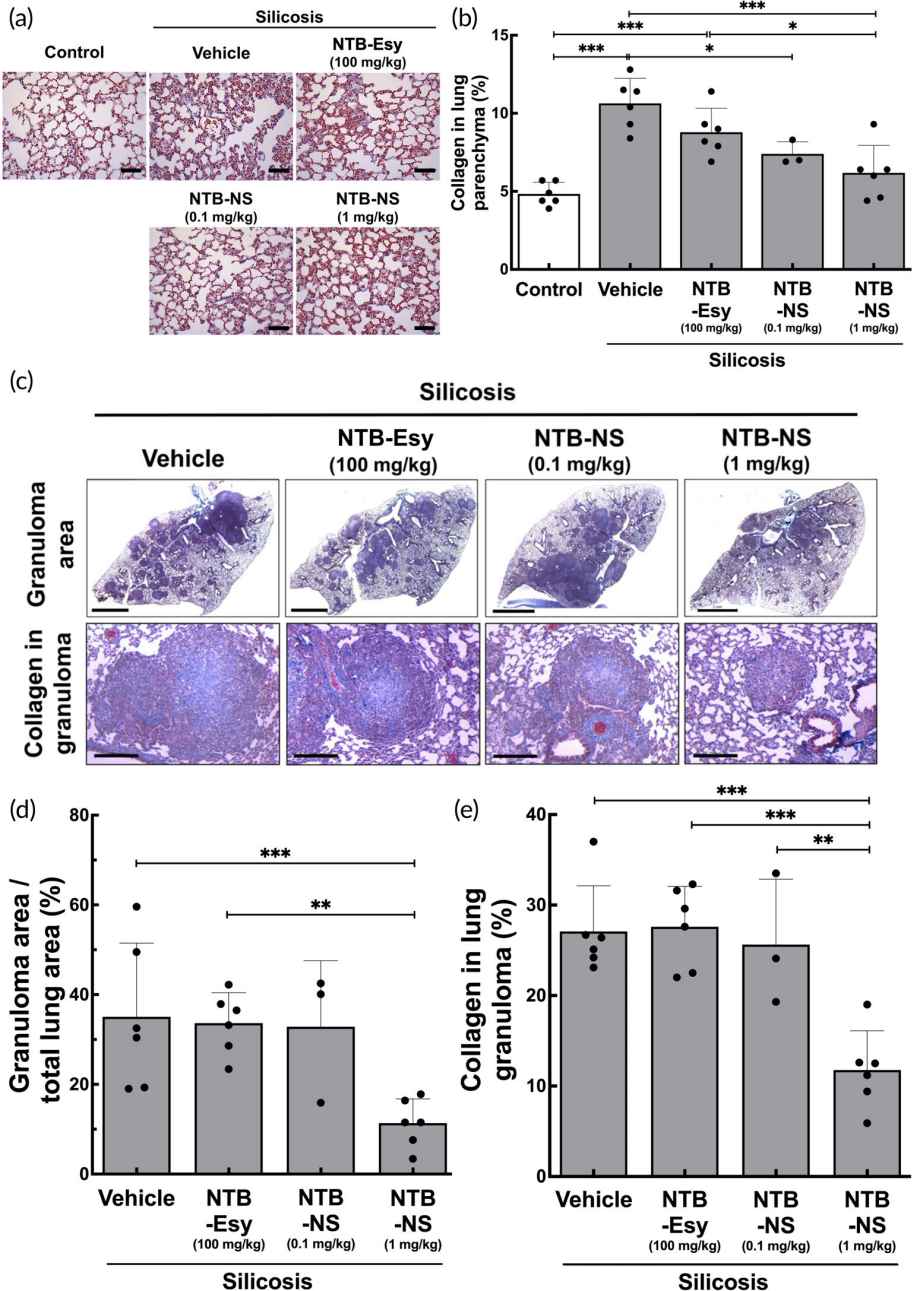
Intratracheally administered NTB‐NS provides significant anti‐fibrotic effect in the lungs of silicotic mice. Silicotic mice received either daily oral dose of NTB‐Esy (100 mg/kg) or intratracheal NTB‐NS at two different NTB doses of 0.1 or 1 mg/kg every 72 hours. (a) Representative histological images and (b) quantification of collagen deposition in alveolar septa of the lung parenchyma (n = 6 mice per group except 0.1 mg/kg NTB‐NS group). Scale bars = 100 μm. (c) Representative histological images of lung parenchyma demonstrating the therapeutic effects of NTB on granuloma area (top: Scale bars = 2 mm) and on collagen deposition in granuloma (bottom: Scale bars = 200 μm). Lung slices were stained with Masson's trichrome to visualize collagen deposition (blue). Quantification of (d) fractional area occupied by granulomas in the lung tissue and of (e) collagen deposition in granulomas (n = 6 mice per group except 0.1 mg/kg NTB‐NS group). Bars represent mean ± SD. The differences are statistically significant as indicated (*P < 0.05, **P < 0.01, ***P < 0.001; one‐way ANOVA followed by a Tukey post hoc test). NTB‐Esy, nintedanib esylate; NTB‐NS, nanosuspension formulation of nintedanib

After completing the treatment regimens, lung tissues were harvested for histopathological analysis where the fibrosis in the lung parenchyma (i.e., alveolar septa) was evaluated as an initial efficacy readout. We found that intratracheal NTB‐NS given at 0.1 or 1 mg/kg NTB dose, unlike 100 mg/kg oral NTB‐Esy, significantly reduced the area of collagen deposition compared to the untreated silicotic animals (*silicosis‐vehicle* group) (*P* < 0.01 or *P* < 0.001, respectively) (Figure 3a,b). Encouragingly, the higher NTB‐NS dose (i.e., 1 mg/kg) near‐normalized the silica‐induced collagen deposition in the alveolar septa (Figure 3b). We next took a closer look at granuloma areas, which are small inflammatory nodules widely observed in the lungs of silicotic patients, particularly those with accelerated silicosis due to very heavy silica exposure.^1^, ^32^, ^33^, ^34^, ^35^, ^36^ Based on our blinded histological analysis (Figure 3c), untreated animals and animals that received oral NTB‐Esy (100 mg/kg, daily) or low‐dose intratracheal NTB‐NS (0.1 mg/kg, every 72 hours) similarly exhibited over 30% granuloma area on average (Figure 3d), suggesting that these treatments were unable to alleviate the granuloma burden. In contrast, the area occupied by granuloma was markedly reduced (~10% on average) in the lungs of animals that received higher intratracheal doses (i.e., 1 mg/kg) of NTB‐NS, resulting in statistically significant differences in comparison to both the untreated (*P* < 0.001) and the oral dosage (*P* < 0.05) groups (Figure 3d). We also found that intratracheal NTB‐NS given at 1 mg/kg roughly halved the collagen fiber deposition within the granuloma on average in comparison to other groups (Figure 3e). The differences were statistically significant compared to all other groups, including untreated animals (*P* < 0.001) and animals treated with either oral NTB‐Esy (*P* < 0.001) or low‐dose intratracheal NTB‐NS (0.1 mg/kg) (*P* < 0.01) (Figure 3e).

**FIGURE 4:**
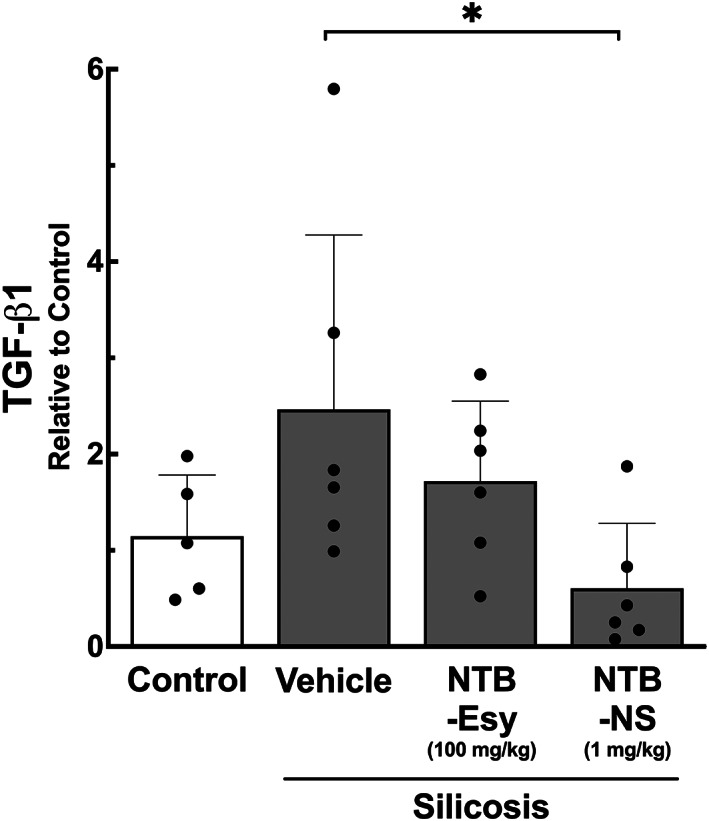
Intratracheally administered NTB‐NS significantly reduces TGF‐β1 expression in the lungs of silicotic mice. The TGF‐β1 mRNA transcript levels in the lung tissues determined by RT‐qPCR. Bars represent mean ± SD (n = 6 mice per group). The difference is statistically significant as indicated (*P < 0.05; one‐way ANOVA followed by a Tukey post hoc test). NTB‐Esy, nintedanib esylate; NTB‐NS, nanosuspension formulation of nintedanib

To further evaluate the anti‐fibrotic effect of locally administered NTB‐NS, we quantified the level of a key pro‐fibrotic mediator, TGF‐β1, in the whole lung homogenates. Upregulation of TGF‐β1, induced by phagocytic uptake of inhaled crystalline silica microparticles, plays a critical role in the formation of silicotic granuloma^5^, ^9^, ^34^, ^37^ and has been validated by post‐mortem examinations of lung tissues from individuals with silicosis.^34^ We excluded 0.1 mg/kg intratracheal NTB‐NS dose here given its limited anti‐fibrotic effect observed in the earlier study (Figure 3). We found that intratracheal NTB‐NS administered at 1 mg/kg every third day, but not the daily oral doses of NTB‐Esy at 100 mg/kg, significantly reduced the mRNA transcript level of TGF‐β1 in the lung tissues (*P* < 0.05, Figure 4). Remarkably, the level was comparable to the homeostatic TGF‐β1 transcript level observed in the lungs of healthy animals (Figure 4). The finding here agrees with the previous in vitro observations with primary human fibroblast that NTB intervenes with TGF‐β signaling and/or with associated pro‐fibrotic events, including myofibroblast differentiation and collagen deposition.^8^, ^38^, ^39^

**FIGURE 5:**
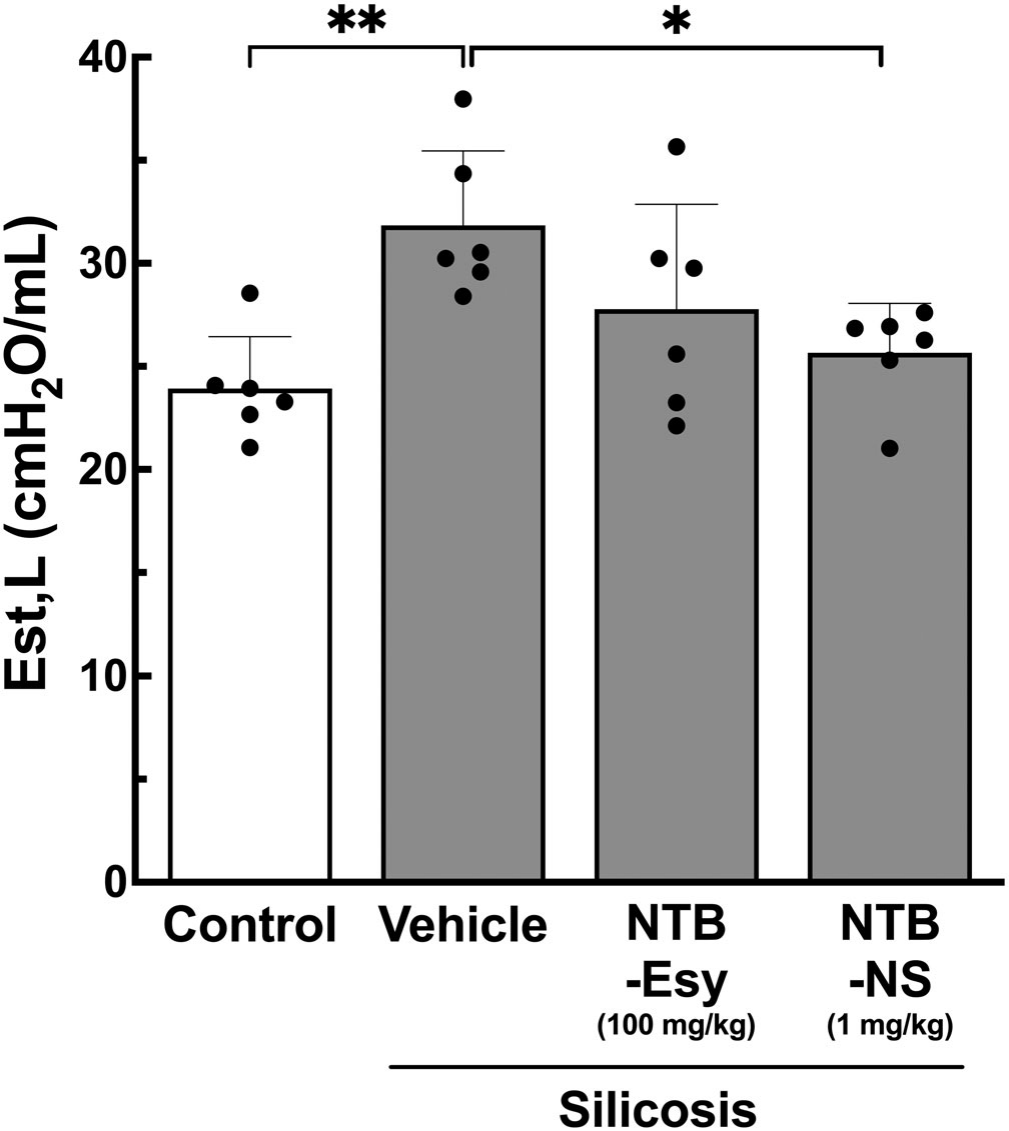
Intratracheally administered NTB‐NS restores mechanical lung function of silicotic mice. Static lung elastance (Est, L). Bars represent mean ± SD (n = 6 mice per group). The differences are statistically significant as indicated (*P < 0.05, **P < 0.01; one‐way ANOVA followed by a Tukey post hoc test). NTB‐Esy, nintedanib esylate; NTB‐NS, nanosuspension formulation of nintedanib

We next went on to test our hypothesis that localized treatment with NTB‐NS would contribute to the normalization of the lung mechanical property, particularly the static lung elastance, based on our observation that NTB‐NS effectively mitigated pulmonary fibrosis in the silicotic lungs (Figures 3 and 4). Elastance is a measure of the pressure required to inflate the lungs and is elevated by pulmonary fibrosis that pathologically transforms the healthy elastic tissue to a scar tissue, as observed in mouse models of silicosis.^28^, ^33^, ^40^, ^41^, ^42^, ^43^ We first confirmed that our silicosis model exhibited a significant elevation of static lung elastance compared to healthy control animals (*control* vs *silicosis‐vehicle*, *P* < 0.01, Figure 5). We then found that intratracheal NTB‐NS (1 mg/kg, every third day), unlike oral NTB‐Esy (100 mg/kg, daily), significantly decreased the static lung elastance (*p* < 0.05) to a level on par with the healthy control animals (Figure 5). Likewise, it has been recently demonstrated that inhaled treatments with NTB‐Esy (2.1 mg/kg, daily), but not daily oral treatments at 30 mg/kg, significantly reduces lung elastance in a mouse model of silica‐induced pulmonary fibrosis.^12^ In contrast to our study, however, the model used in this study did not manifest increased lung elastance over the healthy control animals,^12^ likely indicating a mild or moderate disease phenotype. The discrepancy is most likely attributed to a substantially lower silica dose (2.5 mg/kg) employed to establish their model, compared to our silica dose (800 mg/kg) that has essentially yielded severe silicosis with prevalent granuloma areas.^28^

The robust anti‐fibrotic effects mediated by NTB‐NS were achieved despite more delayed treatment onset, the lower NTB dose, and the reduced dosing frequency implemented in our study compared to the above‐mentioned inhalational NTB‐Esy study.^12^ We first attribute this outcome to sustained drug release from our NS formulation, presumably offsetting the ephemeral nature of NTB in the lung epithelium^12^ to prolong the lung residence time of the drug.^44^, ^45^ Furthermore, nonadhesive surface F127 coating enhances lung retention of our formulation by minimizing the adhesive interactions with airway mucus and lung‐resident macrophages^16^, ^17^, ^18^, ^19^, ^20^ that promote the clearance of inhaled foreign matters from the lung as natural host defense mechanisms.^11^ Of note, we have demonstrated that F127‐coated model NS formulations efficiently traverse human mucus samples harvested from various mucosal surfaces^31^ and that covalent surface shielding of nanoparticles with the hydrophilic segment of F127 (i.e., polyethylene glycol) markedly reduces particle phagocytosis by alveolar macrophages.^46^

Albeit not primarily for inhaled use, there are more than 20 marketed NS products as of 2020.^45^ On the other hand, aqueous drug suspensions are widely used in clinic for the inhaled treatment of patients with numerous lung diseases, including asthma, chronic obstructive pulmonary disease, and cystic fibrosis.^47^, ^48^, ^49^, ^50^ Unlike dry powder formulations, lung deposition of inhaled aqueous drug suspensions is dictated by the aerodynamics of water droplets generated by a nebulizer^51^ and thus fate after lung deposition is the primary consideration for formulation design. To this end, we have focused on enhancing the lung pharmacokinetics by endowing our formulation with the ability to avoid natural clearance mechanisms inevitably encountered following the settlement on lung lumen. Multiple reports have demonstrated that nebulization provides markedly greater or more uniform deposition in human lungs compared to dry powder inhalation.^52^, ^53^ We here show that NTB‐NS can be aerosolized with a vibrating mesh nebulizer without perturbing the physicochemical properties that enable enhanced lung pharmacokinetics. Of note, vibrating mesh nebulizers have been shown in clinical studies to provide superior lung deposition or therapeutic outcomes compared to jet nebulizers which have a longer history of clinical use.^54^, ^55^, ^56^ Importantly, NTB‐NS can be lyophilized for long‐term storage and remote shipping. We thus expect the final product to be a lyophilisate powder that patients can rehydrate with a co‐packaged medical‐grade vehicle solution prior to self‐administration via a portable vibrating mesh nebulizer. The preclinical safety and practical aspects established in this study as well as the relevance of the formulation and drug delivery mode to the current clinical practice collectively underscore the feasibility of implementing our therapeutic approach in clinic.

## 3 EXPERIMENTAL SECTION

### 3.1 Preparation and characterization of NTB‐NS

NTB in a free‐base form (LC Laboratories; Woburn, USA) was dispersed in an aqueous Pluronic F127 (Sigma‐Aldrich, St. Louis, USA) solution at varying NTB and F127 concentrations. This dispersion was then transferred to a tube containing 1.5 g of yttria‐stabilized 0.5 mm zirconium oxide beads (Next Advance, Inc.; Troy, USA), and wet bead‐milling was performed using a TissueLyser LT (Qiagen Inc., Germantown, MD), at a speed of 3000 oscillations/min for 10 hours. Wet milling was performed at 4°C to dissipate heat. Subsequently, NTB‐NS was washed with ultrapure water to remove free NTB and/or F127. All preparation steps were performed using aseptic technique.

Physicochemical properties of NTB‐NS, including particle hydrodynamic diameter, polydispersity index (PDI), and surface charge (i.e., ζ‐potential), were measured using a Zetasizer Nano ZS (Malvern Panalytical; Malvern, United Kingdom) at 90° scattering angle.^57^ Of note, hydrodynamic diameter/PDI and ζ‐potential were measured in ultrapure water and 10 mM NaCl, respectively. The colloidal stability of our formulation was confirmed by monitoring the change of the hydrodynamic diameters of NTB‐NS in ultrapure water or in BALF every 20 minutes up to 6 hours at 37°C. To determine the impact of aerosolization on our formulation, NTB‐NS was diluted in saline at 0.02% (w/v) and aerosolized by a vibrating mesh nebulizer (Aerogen Solo, Chicago, IL) controlled by an Analog Discovery 2 data acquisition device (Digilent, Pullman, WA). Fresh or nebulized NTB‐NS was deposited onto electron microscope grid (EMS Sciences, Hatfield, PA), and particle size and morphology before and after nebulization were evaluated by transmission electron microscopy (Hitachi H7600, Hitachi, Ltd; Tokyo, Japan).

Solid‐state characterization was performed using a LabX XRD‐6100 X‐ray diffractometer (Shimadzu Corp, Kyoto, Japan), operated with 40 kV power and 30 mA current. X‐ray powder diffraction patterns were determined from 3° to 45° on the two theta (2*θ*) scale, at a step size of 20° per second. Encapsulation efficiency was determined by LC‐MS/MS. Briefly, NTB‐NS was fully dissolved in acetonitrile/methanol (2:1, v/v), transferred to autosampler vials and run through the HPLC (Prominence‐i LC‐2030, Shimadzu), equipped with a Phenomenex Luna, C18 (4.6 × 150 mm, 5 μm) column, at room temperature. The water/acetonitrile/trifluoroacetic acid mobile phase (35:65:0.1, v/v/v) was run at isocratic mode for a total of 10 minutes. The column effluent was monitored using a mass‐spectrometric detector (Sciex triple quadrapole 5500 – Sciex, Vaughan, Canada) with electrospray ionization operating in positive mode.

For the sterility assessment, NTB‐NS or F127 solution was applied onto the plate with tryptic soy agar growth medium (Fluka Analytical, St Louis, MO), followed by a 1‐week incubation at 37°C and evaluation of colony formation. Ultrapure water and a suspension of *Pseudomonas aeruginosa* 01 (ATCC27853, 5 × 10^7^ CFU in 200 μL saline) served as negative and positive controls, respectively. To assess the effect of lyophilization‐rehydration on the particle colloidal stability, we lyophilized freshly prepared NTB‐NS in presence or absence of either sucrose or trehalose at a final concentration of 3%. After a 48‐hour freeze‐drying process, NTB‐NS was rehydrated in ultrapure water and analyzed for physicochemical properties using a Zetasizer Nano ZS (Malvern).

### 3.2 Safety assessment of NTB‐NS in healthy mice

#### 3.2.1 Animal treatment

This animal study was approved by the Animal Ethics Committee of the Health Sciences Centre at the Federal University of Rio de Janeiro (process no. 01200.001568/2013‐87, protocol no. 157/19) and the Johns Hopkins University Animal Use and Care Committee (MO19M96). Male 10‐week‐old C57BL/6 mice were anesthetized with sevoflurane, and a 1‐cm‐long midline incision was made to expose the trachea. NTB‐NS at varying doses or vehicle (i.e., ultrapure water) was intratracheally instilled into the mouse lungs using a 30‐gauge needle. The cervical incision was sutured, and mice were returned to their cages. During a period of 24 hours after the injection, we observed whether the animals presented significant body temperature changes, weight loss, and other clinical signs of debilitation, such as piloerection, curved posture, altered respiratory rate, tearing, eyelid changes, dehydration, and reduced locomotor activity.

#### 3.2.2 Evaluation of pro‐inflammatory responses

The influx of inflammatory cells into the airway lumen and alveolar space was quantified by counting cells recovered from BALF. Briefly, BALF was obtained 24 hours after the administration by flushing the airways 2 times with 1 mL of PBS and retrieving the fluid by gentle aspiration. BALF was then centrifuged (239*g*, 10 minutes), and the cell pellet was resuspended in PBS. Subsequently, the cell resuspension was diluted by Türk solution, and total leukocyte population was counted on a Neubauer chamber using an optical microscope. The differential cell counting of polymorphonuclear neutrophils recovered from the BALF was conducted by staining cells using a commercial kit (Panótico Rápido LB, Pinhais, RS, Brazil), followed by calculating the percentage of neutrophils per 100 cells.

After the BALF collection, lung tissues were harvested and fixed with 4% paraformaldehyde in PBS. We then embedded the tissues in paraffin blocks, cut as 4 μm‐thick slices and stained with hematoxylin and eosin. The percentage of neutrophils in alveolar septa was determined by the point‐counting technique, across 10 randomly selected and nonoverlapping microscopic fields.^58^ Histological analyses were performed in a blinded manner.

### 3.3 Therapeutic efficacy assessment of NTB‐NS in preclinical silicosis

#### 3.3.1 Animal treatment

Male 10‐week‐old C57BL/6 mice were randomized in a (healthy) *control group* and a *silicosis group*. To induce silicosis, silica microparticles (0.5‐10 μm in particle diameter, Sigma‐Aldrich) were instilled intratracheally (IT) at a dose of 800 mg/kg using a 30‐gauge needle. *Control group* animals received saline (Figure S3). Fifteen days after the silica instillation, *silicosis group* animals were randomly redistributed in the following four experimental groups: (1) *Vehicle*, daily oral doses of ultrapure water (serving as an untreated control); (2) *NTB‐Esyte*, daily oral doses of 100 mg/kg NTB (serving as a clinically‐relevant control); (3) *NTB‐NS 0.1*, IT instillation every 72 hours at 0.1 mg/kg NTB; (4) *NTB‐NS 1*, IT instillation every 72 hours at 1 mg/kg NTB. Vehicle and NTB‐Esy (LC Laboratories) solution were prepared at 1% hydroxyethyl cellulose prior to administration. Treatments were performed over a period of 18 days.

#### 3.3.2 Lung mechanics analysis

At the end of treatments, mice were sedated with diazepam (1 mg/kg, intraperitoneal), anesthetized with thiopenthal sodium (20 mg/kg, intraperitoneal), tracheotomized, paralyzed with vecuronium bromide (0.005 mg/kg, intravenous), and ventilated with a constant flow ventilator (Samay VR 15, Montevideo, Uruguay) using the following parameters: frequency 100 breaths/min; tidal volume 0.2 mL; fraction of inspired oxygen 0.21. The chest wall was surgically removed and a positive end‐expiratory pressure of 2 cm H_2_O was applied. During a 10‐minute ventilation period, 10 respiratory cycles using the end‐inflation occlusion method were computed for evaluation of lung static elastance (Est, L).^59^, ^60^ Data were analyzed using ANADAT data analysis software (RHT‐InfoData Inc., Montreal Canada).

#### 3.3.3 Histological analysis

Left lung tissues were fixed with 4% paraformaldehyde and embedded in paraffin blocks. We then cut the blocks as 4‐μm thick slices and stained with Masson's trichrome to quantify collagen fiber content.^61^ The fraction areas of collagen fiber in the alveolar septa and granuloma were determined by digital densitometric recognition in ImageJ software (Image‐Pro Plus 5.1 for Windows, Media Cybernetics, Silver Spring, MD).^62^ Airways and blood vessels were carefully avoided during the measurements. Lung sections were also photographed in a microscope (Leica M205 FA, Wetzlar, Germany) to quantify the fractional area occupied by granulomas. Specifically, we captured three images of lung sections at 150‐μm intervals for each animal and subsequently analyzed using ImageJ to measure the areas of individual granulomas and the total lung area. The granuloma fraction was calculated as follows:

$$\text{Granuloma fraction}\;\left(\%\right)=\frac{\sum \left(\text{granulomas area}\right)}{\text{Lung area}}\times 100.$$

#### 3.3.4 RT‐PCR

Right lung tissues were lysed for RNA extraction using the ReliaPrep RNA Miniprep System (Promega Corporation, Madison, WI) as per the manufacturer's protocol. The total RNA concentration and purity was measured by spectrophotometry using a Nanodrop ND‐1000 system (Thermo Fisher Scientific, Waltham, MA). Approximate *A*_260_/*A*_230_ and *A*_260_/*A*_280_ ratios of two were considered ideal for RNA purity.^63^ First‐strand cDNA was synthesized from 1 μg purified RNA using a high‐capacity cDNA reverse transcription kit (Thermo Fisher Scientific). The relative levels of mRNA were measured by SYBR Green detection (Promega) in a PCR Mastercycler Ep Realplex system (Eppendorf, Hamburg, Germany). All samples were measured in triplicate. The relative TGF‐β1 transcript level was calculated as the ratio of the levels of the target gene (i.e., TGF‐β1) over the control gene (ie, acidic ribosomal phosphoprotein P0, 36B4). The primer sequences used in this study were: forward CAACCCAGCTCTGGAGAAAC and reverse GTTCTGAGCTGGCACAGTGA for 36B4; forward CTAATGGTGGACCGCAACAAC and reverse GACAGCCACTCAGGCGTATC for TGF‐β1.

### 3.4 Statistical analysis

Sample size was based on our experience with models of silicosis.^42^, ^43^, ^64^ The normality of the data was confirmed using the Shapiro‐Wilk test and the ROUT test was performed to identify outliers. One‐way analysis of variance (ANOVA) followed by a Tukey post hoc test was then conducted for statistical analysis, and statistical significance was established at *P* < 0.05. All tests were carried out in GraphPad Prism version 9.1.0 (GraphPad Software, San Diego, CA).

## 4 CONCLUSIONS

In the present work, we developed NTB‐NS formulation for localized silicosis treatment. Our formulation possesses characteristics to overcome key extracellular biological delivery barriers, thereby potentially enhancing pharmacokinetic profiles of the payloads in the lung. We demonstrated that intratracheal NTB‐NS provided remarkable anti‐fibrotic activity in a mouse model of silicosis without incurring local and systemic safety concerns. Importantly, the pro‐fibrotic effects were attained in a therapeutic manner (i.e., treatments commenced after the disease was fully established) with a sign of functional normalization and at a 100‐ and a 3‐fold lower dose and dosing frequency, respectively, employed for the oral dosage group that served as a clinically relevant control. To this end, our NTB‐NS formulation constitutes a potential as a novel therapeutic option for localized treatment of silicosis and perhaps other fibrotic lung diseases.

## AUTHOR CONTRIBUTIONS

**Luisa Helena Andrade da Silva:** Conceptualization (equal); data curation (lead); formal analysis (lead); investigation (lead); writing – original draft (equal); writing – review and editing (equal). **Juliana Borges Vieira:** Data curation (supporting); formal analysis (supporting); investigation (supporting). **Marianna Ribeiro Cabral:** Data curation (supporting); formal analysis (supporting); investigation (supporting). **Mariana Alves Antunes:** Data curation (supporting); formal analysis (supporting); investigation (supporting). **Daiheon Lee:** Data curation (supporting); formal analysis (supporting); investigation (supporting). **Fernanda Ferreira Cruz:** Data curation (supporting); investigation (supporting); methodology (supporting); writing – review and editing (supporting). **Justin Hanes:** Project administration (supporting); resources (equal); writing – review and editing (supporting). **Patricia Rieken Macedo Rocco:** Conceptualization (equal); funding acquisition (equal); project administration (equal); resources (equal); supervision (equal); writing – review and editing (equal). **Marcelo Marcos Morales:** Conceptualization (equal); funding acquisition (equal); project administration (equal); resources (equal); supervision (equal); writing – review and editing (equal). **Jung Soo Suk:** Conceptualization (equal); funding acquisition (equal); methodology (equal); project administration (lead); resources (equal); supervision (lead); writing – original draft (equal); writing – review and editing (equal).

### PEER REVIEW

The peer review history for this article is available at https://publons.com/publon/10.1002/btm2.10401.

## Supporting information


**Supplementary Figure 1.** Optimization and characterization of NTB‐NS formulation. (A) Particle size (i.e., hydrodynamic diameter) as a function of Pluronic F127 concentration. NTB was dispersed in F127 solutions at varying concentrations. Data represents average and standard deviation of particle size from three independent samples, measured in triplicate. Differences are statistically significant compared to (†) 1% F127 and (‡) 2% F127 (*P* < 0.05; one‐way ANOVA followed by a Tukey post hoc test). (B) Particle size (i.e., hydrodynamic diameter) as a function of NTB concentration. NTB was suspended at varying concentrations in 1% F127 solution. Data represents average and standard deviation of particle size from three independent samples, measured in triplicate. Differences are statistically significant compared to (†) 5 mg/mL NTB and (‡) 15 mg/mL NTB (*P* < 0.05; one‐way ANOVA followed by a Tukey post hoc test). (C) Representative transmission electron micrograph of the optimized NTB‐NS formulation (i.e., 45 mg/mL NTB and 1% F127). Scale bar = 500 nm. (D) Microbiological analysis of NTB‐NS. No colonies were observed on tryptic soy agar culture dishes, 7 days after inoculation with water, 1% F127, or NTB‐NS, similar to the negative control group but in contrast to the positive control with bacterial culture
**Supplementary Figure 2.** Effect of lyophilization on the physicochemical properties of NTB‐NS. Hydrodynamic diameters (bars) and PDI values (dots) measured before and after lyophilization‐rehydration of NTB‐NS in presence of a disacharride‐based lyoprotectant, either sucrose or trehalose, or without any lyoprotectant (Water). Data represents mean ± SD (n = 5 independent samples). The differences in hydrodynamic diameters are statistically significant as indicated (*P < 0.05, ***P < 0.001; one‐way ANOVA followed by a Tukey post hoc test)
**Supplementary Figure 3.** In vivo safety of NTB‐NS single dose, intratracheally administered in the lungs of healthy mice. (A) Data refers to average and standard deviation of body temperature values, measured in three different time points (n = 4 mice per group). (B) Average and standard deviation of initial (i.e., immediately after the administration) and final (i.e., 24‐hour post‐administration) body weight (n = 4 mice per group). The differences are not statistically significant (one‐way ANOVA followed by a Tukey post hoc test)
**Supplementary Figure 4.** Study design for assessing the therapeutic efficacy of NTB‐NS in a preclinical silicosis model. The silicosis model was established by a single intratracheal instillation of silica microparticles (800 mg/kg) into the lungs of C57BL/6 mice (Silicosis); in parallel, healthy control animals received saline in an identical manner (Control). Fifteen days after the silica instillation, animals were randomly assigned to different groups to receive oral daily doses of NTB‐Esy at 100 mg/kg or intratracheal NTB‐NS at a NTB dose of 0.1 or 1 mg/kg every 72 hours. Lungs were harvested for analysis at Day 34. IT: intratracheal; OR: oral

## Data Availability

The data that support the findings of this study are available from the corresponding author upon reasonable request.
