# Supplementary material for: Development of nintedanib nanosuspension for inhaled treatment of experimental silicosis
Source: Bioeng Transl Med. 2022 Sep 19;8(2):e10401. doi: 10.1002/btm2.10401 (PMC10013831; doi:10.1002/btm2.10401)
Supplement: Supplementary file 1 — Supplementary Figure 1. Optimization and characterization of NTB‐NS formulation. (A) Particle size (i.e., hydrodynamic diameter) as a function of Pluronic F127 concentration. NTB was dispersed in F127 solutions at varying concentrations. Data represents average and standard deviation of particle size from three independent samples, measured in triplicate. Differences are statistically significant compared to (†) 1% F127 and (‡) 2% F127 (P < 0.05; one‐way ANOVA followed by a Tukey post hoc test). (B) Particle size (i.e., hydrodynamic diameter) as a function of NTB concentration. NTB was suspended at varying concentrations in 1% F127 solution. Data represents average and standard deviation of particle size from three independent samples, measured in triplicate. Differences are statistically significant compared to (†) 5 mg/mL NTB and (‡) 15 mg/mL NTB (P < 0.05; one‐way ANOVA followed by a Tukey post hoc test). (C) Representative transmission electron micrograph of the optimized NTB‐NS formulation (i.e., 45 mg/mL NTB and 1% F127). Scale bar = 500 nm. (D) Microbiological analysis of NTB‐NS. No colonies were observed on tryptic soy agar culture dishes, 7 days after inoculation with water, 1% F127, or NTB‐NS, similar to the negative control group but in contrast to the positive control with bacterial culture Supplementary Figure 2. Effect of lyophilization on the physicochemical properties of NTB‐NS. Hydrodynamic diameters (bars) and PDI values (dots) measured before and after lyophilization‐rehydration of NTB‐NS in presence of a disacharride‐based lyoprotectant, either sucrose or trehalose, or without any lyoprotectant (Water). Data represents mean ± SD (n = 5 independent samples). The differences in hydrodynamic diameters are statistically significant as indicated (*P < 0.05, ***P < 0.001; one‐way ANOVA followed by a Tukey post hoc test) Supplementary Figure 3. In vivo safety of NTB‐NS single dose, intratracheally administered in the lungs of healthy mice. (A) Data refe [file BTM2-8-e10401-s001.docx]

Supporting Information

**Development of Nintedanib Nanosuspension for Inhaled Treatment of Experimental Silicosis**

*
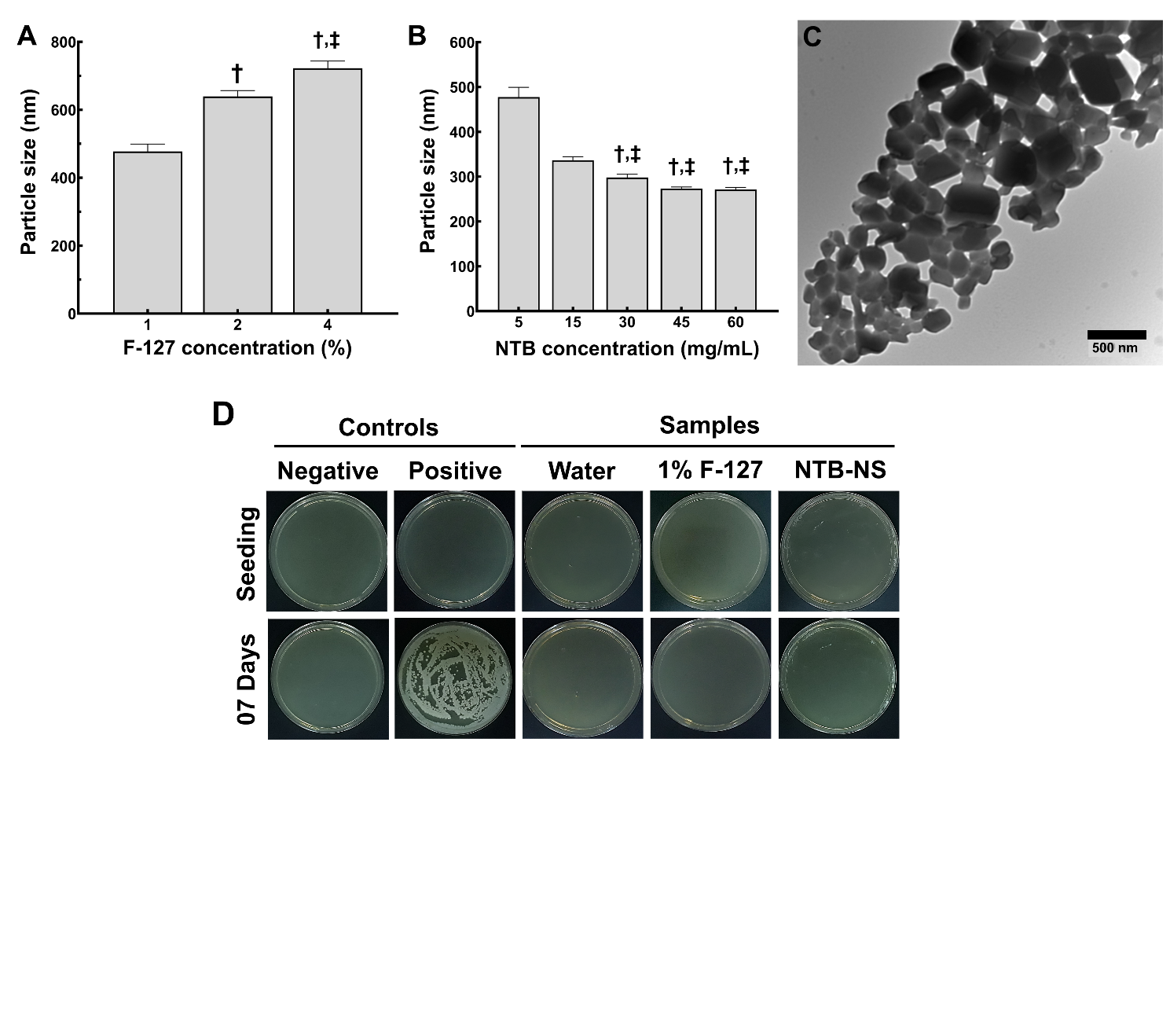
Luisa Helena Andrade da Silva, Juliana Borges Vieira, Marianna Rodrigues Cabral, Mariana Alves Antunes, Daiheon Lee, Fernanda Ferreira Cruz, Justin Hanes, Marcelo Marcos Morales*, Patricia Rieken Macedo Rocco*, and Jung Soo Suk**

**Supplementary Figure 1. Optimization and characterization of NTB-NS formulation.** **(A)** Particle size (*i.e*., hydrodynamic diameter) as a function of Pluronic® F127 concentration. NTB was dispersed in F127 solutions at varying concentrations. Data represents average and standard deviation of particle size from three independent samples, measured in triplicate. Differences are statistically significant compared to (†) 1% F127 and (‡) 2% F127 (*p* < 0.05; one-way ANOVA followed by a Tukey post-hoc test). **(B)** Particle size (*i.e.*, hydrodynamic diameter) as a function of NTB concentration. NTB was suspended at varing concentrations in 1% F127 solution. Data represents average and standard deviation of particle size from three independent samples, measured in triplicate. Differences are statistically significant compared to (†) 5 mg/mL NTB and (‡) 15 mg/mL NTB (*p* < 0.05; one-way ANOVA followed by a Tukey post-hoc test). **(C)** Representative transmission electron micrograph of the optimized NTB-NS formulation (*i.e*., 45 mg/ml NTB and 1% F127). Scale bar = 500 nm. **(D)** Microbiological analysis of NTB-NS. No colonies were observed on tryptic soy agar culture dishes, seven days after inoculation with water, 1% F127, or NTB-NS, similar to the negative control group but in contrast to the positive control with bacterial culture.

**
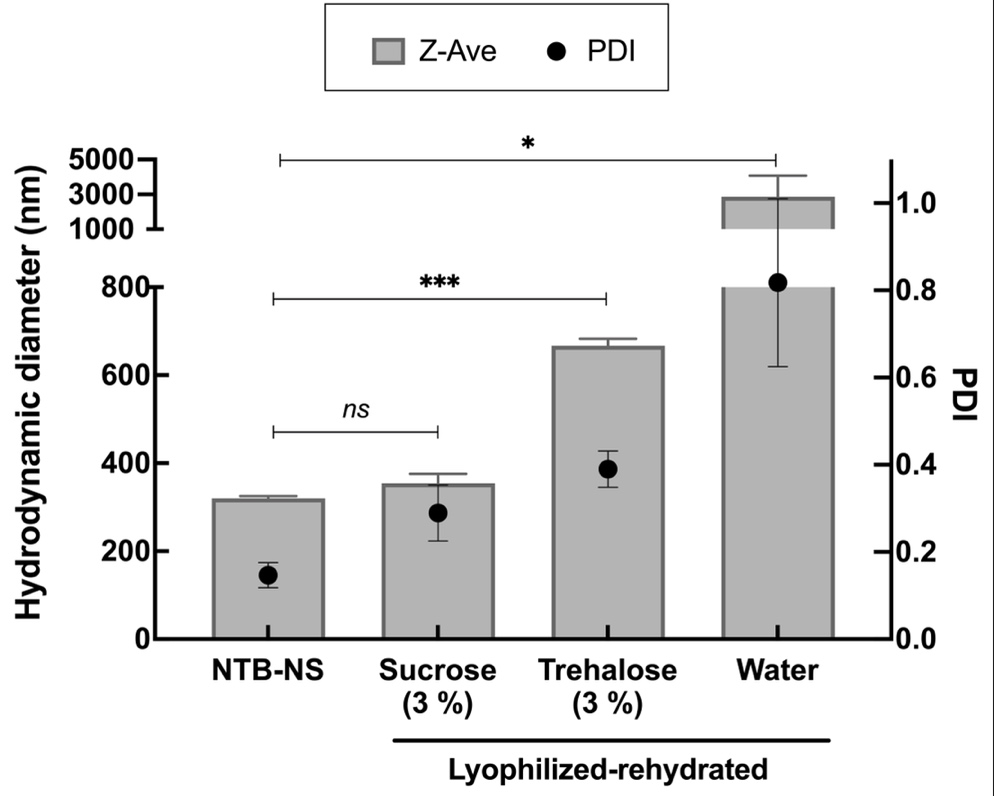
**

**Supplementary Figure 2. Effect of lyophilization on the physicochemical properties of NTB-NS.** Hydrodynamic diameters (bars) and PDI values (dots) measured before and after lyophilization-rehydration of NTB-NS in presence of a disacharride-based lyoprotectant, either sucrose or trehalose, or without any lyoprotectant (Water). Data represents mean ± SD (n = 5 independent samples). The differences in hydrodynamic diameters are statistically significant as indicated (**p* < 0.05, ****p* < 0.001; one-way ANOVA followed by a Tukey post-hoc test).

**
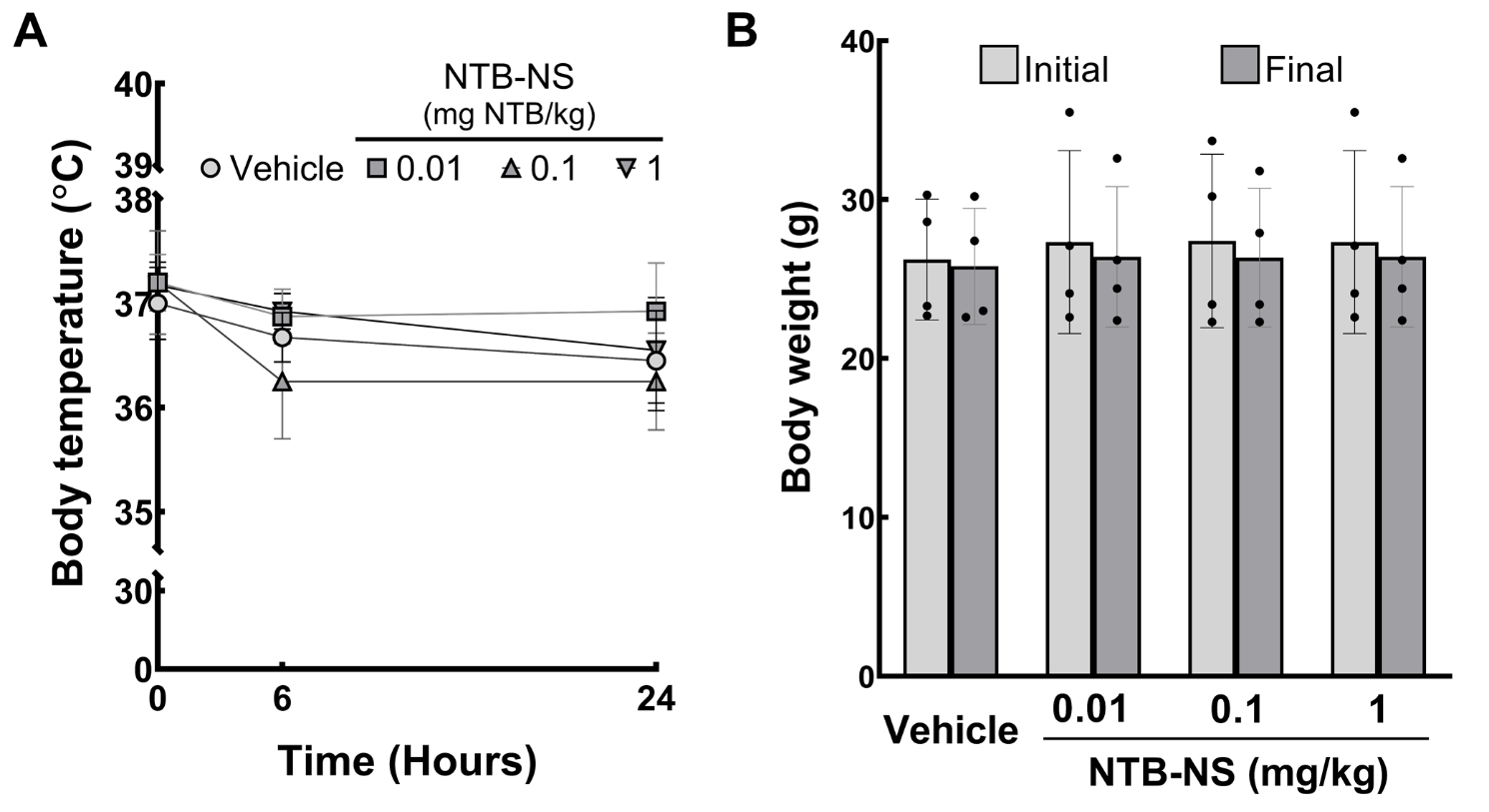
**

**Supplementary Figure 3. In vivo safety of NTB-NS single dose, intratracheally administered in the lungs of healthy mice.** **(A)** Data refers to average and standard deviation of body temperature values, measured in three different time points (n = 4 mice per group). **(B)** Average and standard deviation of initial (i.e., immediately after the administration) and final (i.e., 24-hour post-administration) body weight (n = 4 mice per group). The differences are not statistically significant (one-way ANOVA followed by a Tukey post-hoc test).

**
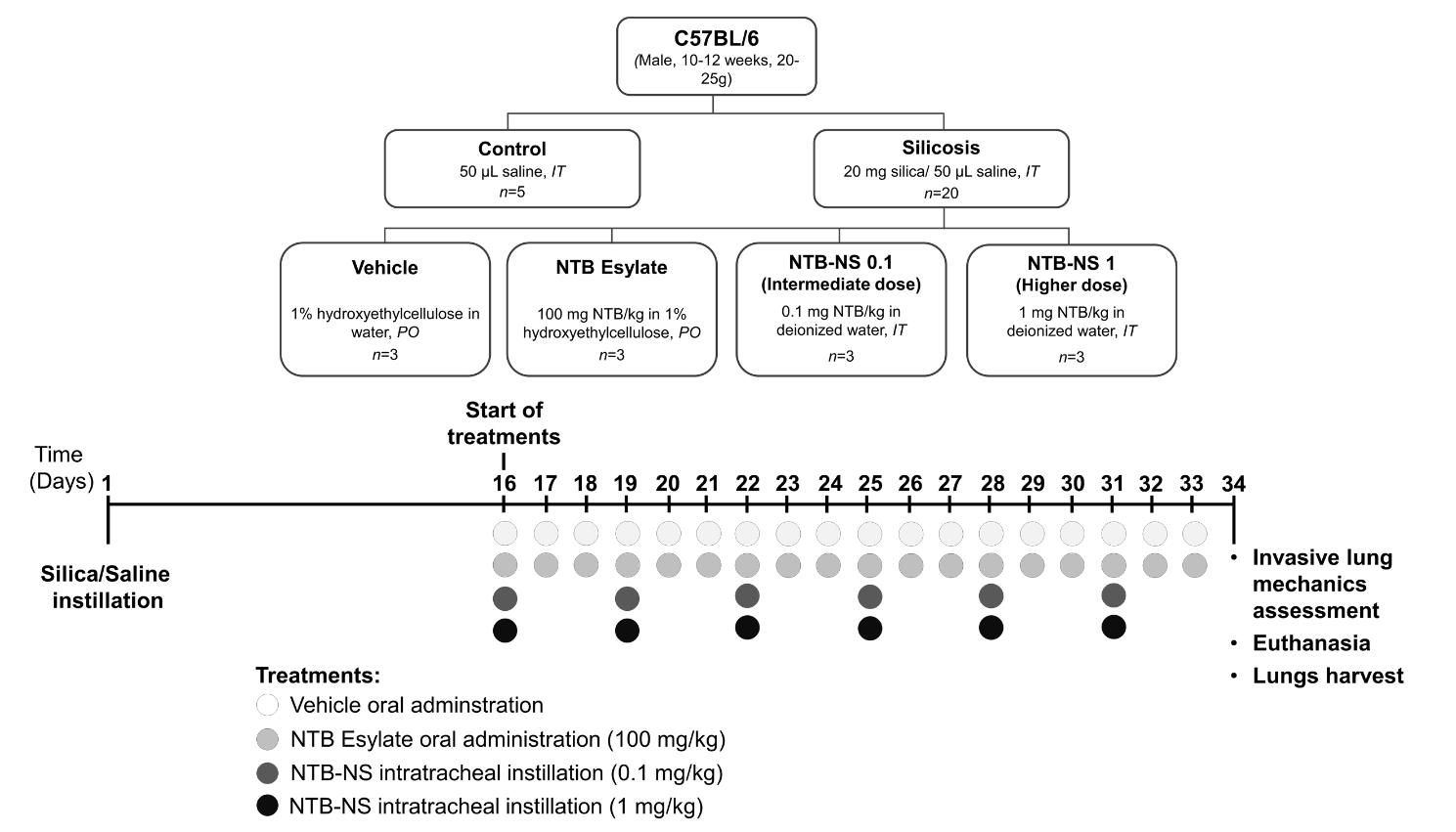
**

**Supplementary Figure 4. Study design for assessing the therapeutic efficacy of NTB-NS in a preclinical silicosis model.** The silicosis model was established by a single intratracheal instillation of silica microparticles (800 mg/kg) into the lungs of C57BL/6 mice (Silicosis); in parallel, healthy control animals received saline in an identical manner (Control). Fifteen days after the silica instillation, animals were randomly assigned to different groups to receive oral daily doses of NTB-Esy at 100 mg/kg or intratracheal NTB-NS at a NTB dose of 0.1 or 1 mg/kg every 72 hours. Lungs were harvested for analysis at Day 34. IT: intratracheal; OR: oral.
